# Supplementary material for: Pediatric Asthma Attack and Home Paint Exposure
Source: Int J Environ Res Public Health. 2021 Apr 13;18(8):4118. doi: 10.3390/ijerph18084118 (PMC8069823; doi:10.3390/ijerph18084118)
Supplement: Supplementary file 1 [file ijerph-18-04118-s001.zip › ijerph-1181479-supplementary.pdf]

Supplementary Material

Pediatric Asthma Attack and Home Paint Exposure

Nadia T. Saif, Julia M. Janecki, Adam Wanner, Andrew A. Colin and Naresh Kumar

Table S1. Demographic and clinical characteristics of pediatric patients in two Miami-based pediatric clinics, N = 163.

Variable	No. of Subjects (%)
Patient Demographics	
Female	84 (52%)
Age (Mean ± SD)	7.85 ± 4.84
0–3 years	32 (20%)
4–7 years	42 (26%)
8–11 years	50 (31%)
12–18 years	37 (23%)
Asthma and Comorbidities	
Diagnosed asthma	36 (22%)
Allergic rhinitis	15 (10%)
Eczema	42 (27%)
Rhinitis symptoms *	43 (26%)
Used anti-allergy medication (past month)	34 (21.8%)
Wheezing ever (among children without asthma)	39 (23.9%)
Wheezing in past year (among children without asthma)	12 (7.4%)
Allergy Diagnosis	
Tested for environmental allergies	35 (22.9%)
Tested positive for any environmental allergen	18 (11.0%)
Cat or dog dander	8 (22.9%)
Mold	5 (14.3%)
Dust mite	11 (31.4%)
Cockroach	5 (14.3%)
Pollen	13 (37.1%)
Home Environment	
Pets in household	79 (48.5%)
Smoker in household	8 (4.9%)
Home interior painted in past year	44 (28.2%)
Air cleaner/purifier in home	49 (33.1%)
Seen cockroach in home (past month)	66 (42.9%)
Seen or smelled mold in home (past month)	8 (4.8%)
Carpet in bedroom	45 (28.5%)
Home construction year	
<1970	35 (25.9%)
1970–1990	47 (34.8%)
1991–present	53 (39.3%)
Home temperature	
60–70	19 (12.7%)
71–75	96 (64.0%)
76+	35 (23.3%)
Temperature used for washing bed sheets	
Cold	50 (32.3%)
Warm	53 (34.2%)
Hot	35 (22.6%)
Parents accessed online pollen or allergy info in past week	20 (14.8%)

* Rhinitis symptoms: sneezing, runny, or blocked nose in absence of cold or flu.

Table S2. Age and gender-adjusted odds of asthma, asthma attack and wheezing with respect to selected covariates (95% confidence interval).

	**Asthma attack**
Age	0.89 (0.73–1.08)
Allergic co-morbidities	
Allergic Rhinitis	1.34 (0.31–5.87
Eczema diagnosis	1.98 (0.47–8.34)
Rhinitis symptoms	Not estimated [a]
Use of anti-allergy medication (past month)	0.98 (0.24–4.09)
Positive for ≥ 1 allergen	1.75 (0.35–8.63)
Has been tested for environmental allergies	2.93 (0.62–13.86)
**Home Environment and Behaviors**	
Smoker in home	0.64 (0.07–5.73)
Pets in home	0.48 (0.11–2.06)
Temperature at which sheets are washed (reference: cold)	
warm	0.50 (0.05–5.25)
hot	4.38 (0.51–37.51)
Home temperature 76+ degrees F (reference: 60–70 F)	2.99 (0.21–41.99)
Parents pay attention to outdoor pollen at least sometimes	0.68 (0.16–2.89)

[a] Odds ratio for rhinitis symptoms and asthma attack could not be estimated, but the relationship was but significant by chi-square association; 13/13 (100%) of patients that had an asthma attack in the past year reported rhinitis symptoms, compared with 13/21 (61.9%) that did not have an asthma attack, $p < 0.05$.

Table S3. Risk of asthma exacerbation (or asthma attack) with respect to paint exposure and potential confounders.

Predictor	Model 1		Model 2		Model 3		Model 4		Model 5		Model 6		Model 7	
	OR	95% CI	OR	95% CI	OR	95% CI	OR	95% CI	OR	95% CI	OR	95% CI	OR	95% CI
Home paint during last one year (1= yes, 0 otherwise)	4.071 *	(0.879–18.868)	6.804 **	(1.093–42.354)	6.944 **	(1.095–44.025)	8.335 **	(1.218–57.045)	8.384 **	(1.196–58.755)	11.238 **	(1.309–96.485)	10.494 **	(1.161–94.851)
Smoker at home (1=yes, 0 otherwise)			0.174	(0.011–2.785)	0.149	(0.008–2.648)	0.154	(0.008–2.931)	0.161	(0.008–3.092)	0.136	(0.007–2.693)	0.155	(0.007–3.357)
Tested positive for allergies (1 = yes, 0 otherwise)					1.574	(0.329–7.537)	0.497	(0.032–7.672)	0.484	(0.029–8.190)	0.713	(0.040–12.825)	0.664	(0.037–12.025)
Multiple allergy status (1 = yes, i.e. two or more, 0 otherwise)							5.027	(0.257–98.408)	4.71	(0.241–92.204)	6.536	(0.297–143.824)	7.506	(0.338–166.684)
Use of antihistamines for asthma and allergy management									1.241	(0.228–6.753)	1.067	(0.183–6.210)	1.107	(0.188–6.512)
Medicine use for asthma and allergy management (1 = yes, 0 otherwise)									0.96	(0.084–10.945)	0.638	(0.048–8.395)	0.61	(0.043–8.676)
Age (year)											0.854	(0.675–1.080)	0.865	(0.678–1.103)
Gender (1 = boys, 2 = girls)													1.876	(0.368–9.557)

** $p < 0.05$, * $p < 0.1$; all models have 36 observations.

Survey Instrument

1. Date of birth (dd/mm/yyyy): **dd/mm/yyyy**

2. Gender: Female ☐ Male ☐

3. Where was your child born?
    - o Florida ☐
    - o Outside Florida, but in U.S. ☐
        - o When did your child move to Florida (month and year)? **mm/yyyy**
    - o Outside U.S. ☐
        - o When did your child move to Florida (month and year)? **mm/yyyy**
        - o

4. Birth place: **name of birth city:______ state:____ 5-digit zip code:_____**
OR, if abroad, country: ______
Current residential address: **city, zip code:________________**

5. Does anyone who lives in your child's home smoke?
    - Yes ☐
    - No ☐
    - Decline to answer ☐

6. Do you have any pets at home?
    - Yes ☐
    - No ☐
    - Decline to answer ☐

7. Has your child **ever** had wheezing or whistling in the chest at any time in the past?
    - Yes ☐
        - o Has your child had wheezing or whistling in the chest in the **past 12 months?**
            - ▪ Yes ☐
            - ▪ No ☐
            - ▪ Don't know ☐
        - o How many attacks of wheezing has your child had in the **past 12 months?**
            - ▪ None ☐
            - ▪ 1 to 3 ☐
            - ▪ 4 to 12 ☐
            - ▪ More than 12 ☐
        - o In the past 12 months, how often, on average, has your child's sleep been disturbed due to wheezing?
            - ▪ Never woken with wheezing ☐
            - ▪ Less than one night per week ☐
            - ▪ One or more nights per week ☐
    - No ☐
    - Don't know ☐

8. In the past 12 months, has your child's chest sounded wheezy during or after physical activity?
    - Yes ☐
    - No ☐
    - Don't know ☐

9. In the past 12 months, has your child had a dry cough at night, apart from a cough associated with a cold or chest infection?

- Yes ☐
- No ☐
- Don't know ☐

10. Has a doctor or other health professional ever told your child that he/she has asthma?
    - Yes ☐
        - How old was he/she when he/she was first told that he/she has asthma? **two-digit year:____**
        - Does he/she still have asthma?
            - Yes ☐
            - No ☐
            - Don't know ☐
        - During the past 12 months, has he/she had an episode of asthma or an asthma attack?
            - Yes ☐
            - No ☐
            - Don't know ☐
        - During the past 12 months, has he/she had to visit an emergency room or urgent care center because of asthma?
            - Yes ☐
            - No ☐
            - Don't know ☐
        - During the past 3 months, has your child taken medication prescribed by a doctor or other health professionals for asthma?
            - Yes ☐
            - No ☐
            - Don't know ☐

      During the past week for how many days {child's name} could not attend school due to worsening of his/her asthma?

      NONE ☐
      1 DAY ☐
      2-3 DAYS ☐
      FULL WEEK ☐
      DON'T KNOW ☐
        - Did he/she develop asthma in Miami?
            - Yes ☐
            - No ☐
            - Don't know ☐
        - Once in Miami, his/her asthma symptoms have:
            - worsened ☐
            - improved ☐
            - not changed ☐
            - don't know
    - No ☐
    - Don't know ☐

11. Has a doctor or other health professional ever told your child that he/she has hay fever?
    - Yes ☐
        - How old was your child when he/she was first told he/she had hay fever? **two-digit year: ____**
        - During the past 12 months, has your child had an episode of hay fever?
            - Yes ☐
            - No ☐
            - Don't know ☐
        - Once in Miami, his/her episodes of hay fever have:

- increased/occur more often ☐
- decreased/occur less often ☐
- not changed ☐
- don't know

- No ☐
- Don't know ☐

12. Has a doctor or other health professional ever told your child that he/she has allergies?
    - Yes ☐
        - How old was your child when he/she was first told he/she had allergies? **two-digit year:** ____
        - During the past 12 months, has your child had any allergy symptoms or an allergy attack?
            - Yes ☐
            - No ☐
            - Don't know ☐
        - Once you moved to Miami, his/her allergy symptoms have:
            - increased in frequency or severity ☐
            - decreased in frequency or severity ☐
            - not changed ☐
            - don't know ☐
    - No ☐
    - Don't Know ☐

13. Has your child been tested for "environmental allergies"?
    - Yes ☐
        - At what age was he/she tested? ________
        - Where was he/she tested?
            - Florida ☐
            - Elsewhere ☐
                - **city/state:**______ OR **country, if not in the U.S.:** ___________
        - Please indicate which allergens, if any, your child was found to be allergic to based on that test (please tick all that apply):
            - ☐ *Alternaria alternata* (a mold)
            - ☐ *Asperigillus fumigatus (a mold)*
            - ☐ Australian pine (*Causarina equisetifolia*)
            - ☐ Bahia grass (*Paspalum notatum*)
            - ☐ Bermuda grass (*Cynodon dactylon*)
            - ☐ *Blomia tropicalis* (a dust mite)
            - ☐ Cat dander
            - ☐ *Cladosporium herbarum (a plant fungus)*
            - ☐ Cockroach
            - ☐ Common ragweed (short; *Ambrosia elatior*)
            - ☐ *Dermatophagoides farinae (a house dust mite)*
            - ☐ *Dermatophagoides pteronyssinus* (a house dust mite)
            - ☐ Dog dander
            - ☐ Elm (*Ulmus americana*)
            - ☐ Maple (box elder; *Acer negindo*)
            - ☐ Mountain cedar (*Juniperus sabinoides*)
            - ☐ Mouse Urine Proteins
            - ☐ Nettle (*Urtica dioica*)
            - ☐ Oak (*Quercus alba*)

        - ☐ *Penicillin*
        - ☐ Rough pigweed (*Amaranthus retroflexus*)
        - ☐ Sheep sorrel (*Rumex acetosella*)
        - ☐ Timothy grass (*Phleum pratense*)
        - ☐ Other: please list ______________________
        - ☐ None
- No ☐
- Don't know ☐

13.a During the past 30 days for how many days did {child's name} take anti-allergy medicines (e.g. Allegra, Zyrtec, Claritin, Benadryl)?

NOT A SINGLE DAY……………………….1
1-2 DAYS……………………………………2
3-5 DAYS……………………………………3
5-10 DAYS…………………………………..4
10-30 DAYS…………………………………5
EVERY DAY………………………………..6
EVERY TWICE...…………………………...7

14. Do you know of any other allergies that your child has (not based on formal testing, but based on experience)?
- Yes ☐
    - Please list these:_________________________
- No ☐
- Don't know ☐

15. In the last 12 months, has a dog, cat, or other small furry animal been removed from your child's home because your child had allergies or asthma?
- Yes ☐
    - What kind of pet was removed from your home? __________
- No ☐

16. Have you avoided bringing new pets into your home because your child had allergies or asthma?
- Yes ☐
- No ☐

17. Has your child **ever** had a problem with sneezing, or a runny, blocked nose when he/she did not have the cold or the flu?
- Yes ☐
    - At what age did this nose problem first occur? **two-digit year:** ________
    - In the **past 12 months**, has your child had a problem with sneezing, or a runny, or blocked nose when he/she did not have the cold or the flu?
        - Yes ☐
        - No ☐
    - In which of the past 12 months did this nose problem occur? (Please tick all tat apply)
        - January ☐
        - February ☐
        - March ☐
        - April ☐
        - May ☐
        - June ☐
        - July ☐
        - August ☐

- September ☐
- October ☐
- November ☐
- December ☐

o Once you moved to Miami, his/her nose symptoms have:
- Increased in frequency or severity ☐
- Increased in frequency or severity ☐
- Not changed ☐
- Don't know ☐

- No ☐
- Don't know ☐

18. Has a doctor or other health professional ever told your child that he/she has eczema?
- Yes ☐
  - How old was your child when he/she was first told he/she has eczema? **two-digit year____**
- No ☐
- Don't Know ☐

19. Has a doctor or other health professional ever told your child that he/she has eczema?
- Yes ☐
  - How old was your child when he/she was first told he/she has eczema? **two-digit year____**
- No ☐
- Don't Know ☐

Q20. Do you pay close attention to the outdoor pollen in your area?

NEVER ☐
RARELY ☐
SOMETIMES ☐
MOST OF THE TIME ☐
ALWAYS ☐

20.a During the past week, how often did you and/or any of the family members access pollen count or allergy index from pollen.com, weather.com or any other local/state/private agency?

NONE ☐
1 DAY ☐
2-3 DAYS ☐
EVERY DAY ☐
TWICE A DAY ☐

20b. How often do you change your air filter in your air conditioning system?

NEVER ☐
EVERY MONTH ☐
EVERY TWO MONTHS ☐
EVERY SIX MONTHS ☐
ONCE A YEAR ☐
DON'T KNOW ☐

20.c Do you have any germicidal air purification system, such as ultraviolet-C, in your air condition system?

YES …..………………………1
NO…….………………………. 2
DON'T KNOW………………..-99

20.d Do you use an air purifier (an electric machine used to clean air) in home?

YES …...………………………1
NO……..………………………. 2
DON'T KNOW………………..-99

20.e Do you ever feel moldy (or must smell) inside your home?

YES …...………………………1
NO……..………………………. 2
NOT SURE…………………..-99

20.f Can you regulate humidity through your air condition/heating system

YES ☐
NO ☐
DON'T KNOW ☐

Q20.g. During the last 12 months, were any areas inside your home painted, such as walls, trim or ceilings?

YES... 1
NO.. 2
REFUSED... 7
DON'T KNOW.. 9

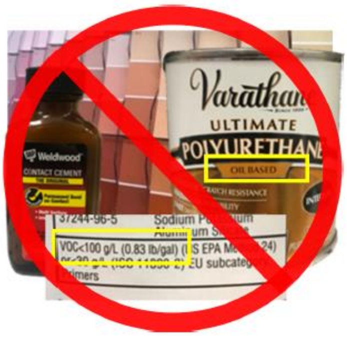

Avoid exposing your child to oil-based paints, paints with color dye, or paints with VOC > 50 g/L.

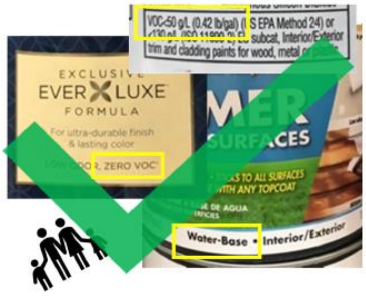

Instead, use water-based without VOCs (also called zero-VOC). If necessary, use low-VOC paint (VOC<50 g/L).

Stay Alert!

Always read the fine print when buying household products and look for **VOC paint concentration** on the label. Be sure to check especially when purchasing paints, colored paints, glues, sealants, spray cans.

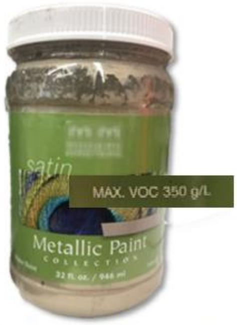

For more information contact the project PI
Dr. Naresh Kumar **nkumar@miami.edu**

UNIVERSITY OF MIAMI
MILLER SCHOOL
of MEDICINE

Protecting Your Children
From the Dangers of Paint Exposure

How to prepare and protect your children with asthma and/or allergies from the hazards of home paint

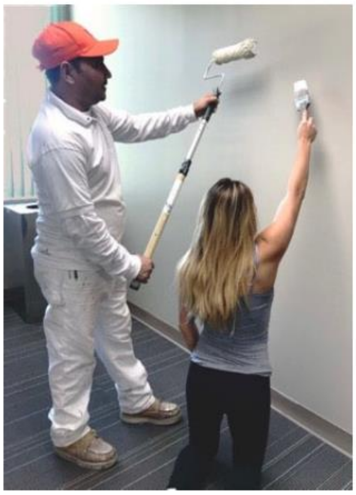

Supported by
The National Institute of Health

WHAT ARE THE DANGERS OF PAINT EXPOSURE?

Giving your walls a fresh coat of paint is a basic part of home upkeep. Many household products, including most paints, release substances called **volatile organic compounds (VOCs)** are released as gases from certain solids or liquids. Most of these products contain adhesives (like glue), which are a common source of VOCs.

WHICH HOUSEHOLD PRODUCTS MAY CONTAIN VOCs?

- Oil-based paint
- Paint with added color dye
- Wood preservatives
- Aerosol sprays
- Cleaners/disinfectants
- Adhesives
- Air fresheners
- Furniture fabrics

Paints with VOCs can be harmful to yourself and your children. VOCs have toxic effects on your brain, immune, and respiratory systems. Short term effects can include headaches, dizziness, and memory and vision problems. **Children with asthma or allergies are most at risk to these harmful effects if exposed to VOCs for long periods of time.**

HOW DOES PAINT AFFECT ASTHMA AND ALLERGIES?

Freshly painted walls release VOCs while they dry, and also for several weeks after. Continuously breathing in these chemicals can cause inflammation in your children's airways and lungs. Over time, your body produces more mucus to fight this inflammation, which can make it harder to breathe and fight infections. This can lead to the worsening of your child's existing asthma or allergy symptoms. In some cases, exposure to VOCs can lead to the development of asthma, rhinitis, or eczema.

Using zero-VOC, water-based paint is the only way to ensure these chemicals aren't in your paint. Even if your base paint is zero-VOC, be careful: adding color can be harmful because color dye

HOW TO PROTECT YOUR CHILDREN FROM HARMFUL PAINT EXPOSURE

ELIMINATE

- **Use *zero-VOC* water-based paints**
- **Do not add color dye to paints**

AVOID

- **Keep children with asthma and/or allergies away from freshly painted areas for 2 to 4 weeks**

REDUCE

- **Use *low-VOC* water-based paints**
- **Use an air purifier that can reduce VOCs (ex. filter with activated carbon)**
- **Ventilate household for weeks after painting**

PREPARE AND PROTECT

- **Before you paint, consult your child's doctor about medicines to manage allergies and/or asthma**
